# Validation of the Spanish versions of the long (26 items) and short (12 items) forms of the Self-Compassion Scale (SCS)

**DOI:** 10.1186/1477-7525-12-4

**Published:** 2014-01-10

**Authors:** Javier Garcia-Campayo, Mayte Navarro-Gil, Eva Andrés, Jesús Montero-Marin, Lorena López-Artal, Marcelo Marcos Piva Demarzo

**Affiliations:** 1 Department of Psychiatry, Miguel Servet Hospital, Aragon Institute of Health Sciences (I + CS), Zaragoza, Spain; 2 Primary Care Prevention and Health Promotion Research Network (RedIAPP), Zaragoza, Spain; 3 Bit&Brain Technologies, Zaragoza, Spain; 4 12 de Octubre Research Institute, Madrid, Spain; 5 Faculty of Health Sciences and Sports, University of Zaragoza, Huesca, Spain; 6 Lozano Blesa University Clinic Hospital, Zaragoza, Spain; 7 Center for Mindfulness and Health Promotion, Department of Preventive Medicine, Escola Paulista de Medicina, Universidade Federal de São Paulo (UNIFESP), São Paulo, Brazil

**Keywords:** Self-compassion, Validation, Spanish, Mindfulness

## Abstract

**Background:**

Self-compassion is a key psychological construct for assessing clinical outcomes in mindfulness-based interventions. The aim of this study was to validate the Spanish versions of the long (26 item) and short (12 item) forms of the Self-Compassion Scale (SCS).

**Methods:**

The translated Spanish versions of both subscales were administered to two independent samples: Sample 1 was comprised of university students (n = 268) who were recruited to validate the long form, and Sample 2 was comprised of Aragon Health Service workers (n = 271) who were recruited to validate the short form. In addition to SCS, the Mindful Attention Awareness Scale (MAAS), the State-Trait Anxiety Inventory–Trait (STAI-T), the Beck Depression Inventory (BDI) and the Perceived Stress Questionnaire (PSQ) were administered. Construct validity, internal consistency, test-retest reliability and convergent validity were tested.

**Results:**

The Confirmatory Factor Analysis (CFA) of the long and short forms of the SCS confirmed the original six-factor model in both scales, showing goodness of fit. Cronbach’s α for the 26 item SCS was 0.87 (95% CI = 0.85-0.90) and ranged between 0.72 and 0.79 for the 6 subscales. Cronbach’s α for the 12-item SCS was 0.85 (95% CI = 0.81-0.88) and ranged between 0.71 and 0.77 for the 6 subscales. The long (26-item) form of the SCS showed a test-retest coefficient of 0.92 (95% CI = 0.89–0.94). The Intraclass Correlation (ICC) for the 6 subscales ranged from 0.84 to 0.93. The short (12-item) form of the SCS showed a test-retest coefficient of 0.89 (95% CI: 0.87-0.93). The ICC for the 6 subscales ranged from 0.79 to 0.91. The long and short forms of the SCS exhibited a significant negative correlation with the BDI, the STAI and the PSQ, and a significant positive correlation with the MAAS. The correlation between the total score of the long and short SCS form was *r* = 0.92.

**Conclusion:**

The Spanish versions of the long (26-item) and short (12-item) forms of the SCS are valid and reliable instruments for the evaluation of self-compassion among the general population. These results substantiate the use of this scale in research and clinical practice.

## Background

Mindfulness has been defined as a quality of consciousness involving accepting and non-judgmental present-centred attention and awareness [1]. Meta-analysis suggests that mindfulness-based interventions (MBIs) are efficacious in treating many psychological and somatic disorders, such as anxiety and mood disorders [2]. Both mindfulness [3] and other higher order cognitive processes (including changes in meta-cognition, attentional allocation or directed awareness), which mediate the effects of mindfulness, are considered difficult to assess [4].

Positive mental states are a commonly associated with MBIs. These states might include the attitudes with which one approaches things (e.g., acceptance) [5] or the approach that one takes to interpret private experiences (e.g., self-compassion) [6]. Several studies have demonstrated that MBIs increase positive emotions [7], and that positive emotions also foster positive outcomes for MBIs [8].

In recent years, one of the most studied positive mental states associated with mindfulness is self-compassion. The newfound interest in this positive mental state could reflect the fact that 1.- Self-compassion has been one of the scarce theory-consistent constructs shown to mediate change in MBIs [8]; 2.- Self-compassion provides a better explanation of the clinical outcomes in anxiety and depression [9]; and 3.- Self-compassion is a well-defined construct [10] that is less difficult to assess than mindfulness.

Self-compassion has been defined as “being touched by and open to one’s own suffering, not avoiding or disconnecting from it, generating the desire to alleviate one’s suffering and to heal oneself with kindness” [10]. There are three theoretical facets to self-compassion, represented by pairs of opposing subscales and identified by the positive quality of these facets: 1.- Self-kindness and self-judgment; 2.- Common humanity and isolation; and 3.- Mindfulness and over-identification [11]. The self-kindness facet represents an alternative to self-criticism and blaming, and clinical characteristics associated with depression. Common humanity acknowledges that human suffering is inherent to the nature of life and intimately associated with the suffering of others. This facet might be associated with general wellbeing [10]. Moreover, the mindfulness facet represents a stance of equanimity, rather than over-identification, towards difficult and uncomfortable thoughts and experiences [10,11].

In Buddhist tradition, wisdom (a quality only attainable through mindfulness) and compassion are considered complementary and inseparable. Compassion includes mindfulness as one of its three component facets. And mindfulness is also based on compassion because it implies not only teaching our minds to focus and maintain contact with whatever is going on in the present moment, but also to apply the skills of kindness, patience and generosity to a body and mind that are always, at least partially, beyond our own control [12].

Both mindfulness and self-compassion are constructs that describe how individuals relate to themselves and to their experiences, including unpleasant experiences such as pain and fear. In their current operative definitions [3,10] mindfulness and self-compassion are highly correlated, and a correlation coefficient of *r* = 0.69 has been reported in a nonclinical population [13]”.

Self-compassion is assessed using the Self-Compassion Scale [10]. The original SCS contains 26 items, measuring six components of self-compassion: Self-Kindness, Self-Judgment, Common Humanity, Isolation, Mindfulness and Over-Identification [10]. Adequate psychometric properties are also reported [10]. The items are rated on a five-point response scale ranging from 1 (almost never) to 5 (almost always). Recently, a shorter version (12 items) of the SCS has been developed [14]. The aim of this study was to validate and assess the psychometric properties of the Spanish versions of both questionnaires (long and short forms). The reason for validating both questionnaires simultaneously was that none of them had previously been validated in Spanish. The validation of the short form required the calculation of the correlation with the validated long form, and therefore it seemed logical to validate both at the same time. On the other hand, the simultaneous validation of both instruments allowed them to be compared and the usefulness of each one to be identified. This facilitates the work of researchers by allowing them to obtain all the information in a single manuscript.

## Methods

### Participants

We recruited two different samples to validate both questionnaires independently. The long questionnaire (26-item) form of the SCS was administered to a random sample of health sciences students recruited from the University of Zaragoza, Spain (sample 1); and the short (12-item) form was administered to a random sample of workers recruited from the Aragon Health Service (Study 2). The long form was also included in the protocol for this second group to calculate the correlation between the short and long forms of the SCS.

The following inclusion criteria were used: Individuals ranging in age from 18 to 65 years, who agreed to participate in this study. The exclusion criteria included any medical or psychiatric disorders that would impede the individual from answering the questionnaire correctly and poor knowledge of the Spanish language. The sample size was calculated according to the recommended 10:1 ratio for the number of subjects to the number of test items [15]. The questionnaires and protocols used in this study were approved through the Ethical Committee of the regional health authority (21/2012; 28th Nov 2012), and the patients signed a consent form attesting to their willingness to participate in this study. The study was conducted between September 2012 and May 2013.

### Procedure

Regarding procedure, both samples were informed about the study by means of announcements posted on noticeboards, both in the different faculties (sample 1) and in the hospitals where the study was carried out. In a second phase, randomized sampling was carried out based on the final list of the individuals included in the two samples. For sample 1 (health sciences students from the University of Zaragoza) a simple randomized sampling was used. For sample 2 (workers recruited from the Aragon Health Service) a conglomerate sampling was utilized, with each of the included hospitals considered as a conglomerate. Participants who refused to participate were substituted with other participant with similar characteristics. Recruitment continued up to the required sample was completed.

Questionnaires were administered by a research psychologist. Each of the participants was presented with an informed consent form, which included the aims of the study, the advantages/disadvantages of participating, and notification that the data would be processed anonymously. Participants received no compensation for their participation.

### Measures

#### Background variables

The background information obtained from the participants included age, sex, level of education (primary school, secondary school, university), ethnicity and meditation practice.

#### Self-Compassion Scale (SCS)

The SCS [11] is a 26-item questionnaire designed to assess overall self-compassion (total score) and components of self-compassions across three conceptually distinct, but theoretically related, facets: common humanity (SCS-CH), mindfulness (SCS-M), and self-kindness (SCS-SK). Although the construct was defined using these three facets [10], the factor analysis suggested six subscales, representing positive and negative aspects of each facet [11]. These items were designed to assess how respondents perceive their actions toward themselves in difficult times and are rated using a Likert-type scale anchored from 1 (almost never) to 5 (almost always). The SCS has adequate reliability and validity [11], even in different cultures [16].

A short form (12 items) of the SCS had recently been developed [14]. The SCS–SF demonstrated adequate internal consistency (Cronbach’s alpha ≥ 0.86) and a near-perfect correlation with the long form of the SCS (r ≥ 0.97 all samples). Confirmatory factor analysis on the SCS–SF supported the same six-factor structure as used in the long form.

#### Mindful Attention Awareness Scale (MAAS)

The MAAS [17] is a 15-item measure of mindfulness. The item content was designed to reflect the opposite of the construct of mindfulness, or “mindlessness,” thus endorsing the item content at a lower frequency suggests a higher level of mindfulness. Each item is rated on a scale from 1 (almost always) to 6 (almost never) in relation to the respondent’s “everyday experience,” and there is no specified time frame for these ratings. The item ratings are averaged to form the total score. This scale has been validated in Spanish and shows adequate psychometric parameters [18].

#### State-Trait Anxiety Inventory–Trait (STAI)

The Spielberger State-Trait Anxiety Inventory–Trait form [19] is a commonly used self-reported 20-item anxiety questionnaire used in both general populations and patients. This questionnaire is based on the following 4-point Likert-type scale: 0: almost never; 1: sometimes; 2: often; and 3: almost always. This assessment shows adequate psychometric properties and has been validated in Spanish [20].

#### Beck Depression Inventory (BDI)

The Beck Depression Inventory [21,22] is a self-reported 21-item questionnaire, in which each answer is scored on a scale of 0 to 3. This assessment is one of the most widely used questionnaires designed to examine depression severity and to characterise the cognitive, affective, motivational and somatic symptoms of depression. The standard cut-offs include 0–9: minimal depression; 10–18: mild depression; 19–29: moderate depression; and 30–63: severe depression. The BDI shows adequate psychometric properties and has been validated in Spanish [23].

#### Perceived Stress Questionnaire (PSQ)

The PSQ is specifically designed to measure stress in clinical psychosomatic research. This test consists of 30 items, to which the respondents rate how often an item applies to them using a 4-point scale (1: almost never; 2: sometimes; 3: often; and 4: usually). The validation study for this questionnaire showed excellent psychometric properties [24], and it has been used in research, demonstrating an adequate predictive value in stress-related diseases, such as ulcerative colitis [25]. The PSQ index was obtained according to the indications, i.e., PSQ = (raw score 30) / 90, previously described in Levenstein et al. [25]. The validated Spanish version of this assessment was used [26].

### Validation process

Permission to translate and validate the SCS was obtained from the original authors [11,14]. Two researchers, unaware of the objectives of the questionnaire, provided the initial Spanish translation. Each researcher translated the questionnaire separately. Subsequently, two bilingual linguistics experts, with no specific knowledge regarding the instruments, performed back-translations. Moreover, a native English-speaking teacher determined that the two English versions of this assessment were equivalent. Any differences between the translations were resolved through mutual agreement. Both translators and authors were present during the agreement. The authors are familiar with written technical English and the psychological construct assessed using the questionnaire. The usual guidelines were followed for cross-cultural adaptations [27]. This paper is part of a broader research endeavour on the psychological and neurobiological aspects of mindfulness [28,29]. The final Spanish versions of both questionnaires are shown in Additional file 1: Annex 1 and 2.

The assessments were performed at two different points over a 1–2 week interval. There is no evidence available to aid in the selection of the time interval between the administrations of the questionnaire to assess the test-retest reliability of health status instruments [30]. This time interval was selected because it was too short for clinical changes to occur. The subsample for the second assessment, used to measure test-retest reliability, was randomly selected.

### Statistical analyses

The demographic data were analysed using the descriptive statistics of mean, standard deviation (SD) and range. Prior to conducting the statistical analyses, we examined the data for univariate and multivariate outliers. To detect the presence of univariate outliers, the frequency distributions of each item were examined (values ≥ 3 standard deviations from the mean indicate univariate outliers). The multivariate outliers were screened using the Mahalanobis distance scores for all cases (D2). A D2 probability ≤ 0.01 indicates the existence of multivariate outliers [31]. We did not detect any outliers; therefore, all cases were retained for the statistical analyses.

We used Confirmatory Factor Analysis (CFA) to analyse the dimensionality of the SCS short and long forms. We proposed the previously described six-factor model [11,14]. EQS software for Windows version 6.1 [32] was used to conduct the CFA. The maximum likelihood with a robust correction method was used to adjust for distributional problems in the data set. Although a model with a non-significant chi-square estimate is generally considered a model with good fit, Hu and Bentler [33] recommended combinational rules to evaluate the model fit. Therefore, we analysed the following indices (values in parentheses denote goodness of fit standards): Comparative Fit Index and Goodness of Fit Index (CFI and GFI > .90) and Root Mean Square Error of Approximation (SRMR) and Standardised Root Mean-Square Residual (RMSEA < .08) [34]. We selected these statistics to measure the fit because previous studies have validated the performance and stability of these tests [35].

We examined the internal consistency, test-retest and construct validity of the SCS. Cronbach’s α coefficient [36] was used to analyse the internal consistency of the scale. Corrected item-total correlations, in which an item is correlated with the total scale score, excluding itself, were tested for each item. The consistency of the SCS total score over time (test-retest reliability) was assessed using the Intra-class Correlation Coefficient (ICC). The construct validity was examined by correlating the SCS with theoretically related and unrelated constructs. Pearson’s *r* correlations were performed to evaluate univariate relationships between the SCS and the following variables: mindfulness, depression, anxiety and perceived stress. We used the effect size criteria outlined by Cohen [37] to evaluate the substantive significance of the correlations (i.e., large correlations are those > .50, medium correlations ranging from 0.30 to 0.49, and small correlations ranging from 0.10 to 0.29).

## Results

### Characteristics of the sample

For sample 1 (for the validation of the long form of SCS questionnaire), we recruited 273 health sciences students attending the University of Zaragoza. The students were randomly selected from the university lists. Of these, 3 (0.01%) individuals refused to participate and 2 (0.007%) individuals were ruled out because they were not fluent in Spanish. The characteristics of the final sample (N = 268) included 59.7% females, a mean age of 20.54 years (SD = 2.11), 99.2% Europeans and 1.8% of these individuals practiced any type of meditation at least once a month. These subjects studied medicine (57.08%), nursing (26.11%) and physiotherapy (16.81%).

For sample 2 (for the validation of the short form of the SCS questionnaire), a total of 283 individuals working for the Aragon Health Service were recruited. They were randomly selected from the list of doctor and nurses working in ten health centres, representing different areas of the city of Zaragoza. Of these, 11 (3.8%) individuals refused to participate and 1 (0.003%) individual was ruled out because he was not fluent in Spanish. The characteristics of the final sample (N = 271) included 67.89% females, a mean age of 38.43 years (SD = 9.67), 96.6% Europeans and only 2.95% of these individuals practiced any type of meditation at least once a month. All subjects were doctors (63.1%) and nurses (36.9%).

### Description of the variables

All items were examined in terms of mean, standard deviation, skewness and kurtosis. Univariate values approaching at least 2.0 for skewness and 7.0 for kurtosis indicate marked non-normality [29]. On the basis of these values, all data showed normality. Table 1 summarises the descriptive statistics (mean and standard deviation) of the questionnaires used in both samples included in this study.

**Table 1:** Means and standard deviations of the results obtained from the questionnaires in both sample groups used in this study

	**Sample 1 (Long form)**	**Sample 2 (Short form)**
	**N = 268**	**N = 271**
**Questionnaires used (range)**	**Mean (SD)**	**Mean (SD)**
Self-compassion scale*(6–30)	17.95 (3.68)	16.89 (3.46)
Self-kindness (1–5)	3.14 (.68)	2.94 (.71)
Self-judgment (1–5)	3.02 (.71)	2.91 (.65)
Common humanity (1–5)	2.91 (.65)	3.12 (.73)
Isolation (1–5)	2.87 (.72)	2.67 (.61)
Mindfulness (1–5)	3.18 (.74)	2.95 (.68)
Over-identification (1–5)	3.14 (.77)	2.91 (.83)
MAAS (1–6)	4.12 (.62)	4.01 (.59)
STAI (0–60)	20.64 (8.23)	22.36 (7.84)
BDI (0–63)	5.26 (2.12)	6.04 (1.86)
PSQ (0–1)	.31 (.14)	.37 (.17)

*Overall self-compassion scores (in both the long and short forms) were calculated after reverse coding the self-judgment, isolation and over-identification items, followed by the summation of the six subscale means.

### Confirmatory Factor Analysis (CFA)

The CFA of the long form (26 items) of the SCS in sample 1 confirmed that the original six-factor model [11] showed good fit indices: CFI = .95, GFI = .93, SRMR = .05, RMSEA = .06 [.05-.08]. In sample 2, the CFA of the short form of the SCS demonstrated that the original six-factor model [12] also showed goodness of fit: CFI = 0.94; GFI = 0.91; SRMR = 0.05; RMSA = 0.07 (0.05-0.08)]. The factor loadings of the 26 items of the long form and the 12 items of the short form of the SCS are summarised in Table 2.

**Table 2:** Items and Factor Loadings for the Six Subscale Factors of the long and short forms of the Self-Compassion Scale (SCS)

**Item**	**(Numbers as in the long form SCS)**	**Loading loading**
	**Long-form**	**Short-form**
Self-kindness subscale		
5.- I try to love myself when I’m feeling emotional pain.	.68	--
12.- When I’m going through a very hard time, I give myself the care and tenderness I need.	.72	.66
19.- I’m kind to myself when I’m experiencing suffering.	.70	--
23.- I’m tolerant of my own flaws and inadequacies.	.71	--
26.- I try to be understanding and patient towards aspects of my personality that I don’t like.	.69	.71
Self-judgment subscale		
1.- I’m disapproving and judgmental of my flaws and inadequacies.	.70	.67
8.- When times are really difficult, I tend to be tough on myself.	.67	--
11.- I’m intolerant and impatient towards aspects of my personality that I don’t like.	.62	.65
16.- When I see aspects of myself that I don’t like, I am down on myself.	.77	--
21.- I can be a bit cold-hearted towards myself when I’m experiencing suffering.	.68	--
Common humanity subscale		
3.- When things are going badly for me, I see the difficulties as part of life that everyone goes through.	.61	--
7.- When I’m down and out, I remind myself that there many other people in the world share these same feelings.	.71	--
10.- When I feel inadequate in some way, I try to remind myself that feelings of inadequacy are shared by most people.	.72	.74
15.- I try to see my failures as part of the human condition.	.68	.63
Isolation subscale		
4.- When I think about my inadequacies, I tend to feel more separate and cut off from the rest of the world.	.69	--
13.- When I’m feeling down, I tend to feel like most other people are happier than I am.	.62	.59
18.- When I’m really struggling, I tend to feel like other people must be having an easier time.	.69	--
25.- When I fail at something that’s important to me, I tend to feel alone in my failure.	.70	.63
Mindfulness subscale		
9.- When something upsets me, I try to keep my emotions in balance.	.61	.64
14.- When something painful happens, I try to take a balanced view of the situation.	.71	.64
17.- When I fail at something important to me, I try to keep things in perspective.	.72	--
22.- When I’m feeling down, I try to approach my feelings with curiosity and openness.	.68	--
Over-identification subscale		
2.- When I’m feeling down, I tend to obsess and fixate on everything that is going wrong.	.72	.65
6.- When I fail at something important to me, I become consumed by feelings of inadequacy.	.67	.63
20.- When something upsets me, I get carried away with my feelings.	.62	--
24.- When something painful happens, I tend to exaggerate the incident.	.71	--

### Internal consistency and test-retest reliability

Cronbach’s α for the 26-item SCS was 0.87 (95% CI = 0.85-0.90), ranging from 0.72 to 0.79 for the 6 subscales. Cronbach’s α for the 12-item SCS was 0.85 (95%CI = 0.81-0.88), ranging from 0.71 to 0.77 for the 6 subscales. All corrected item-total *r* correlation coefficients were above 0.30: the scoring of the items of the 26-item SCS ranged between 0.41 and 0.68, and for the 12-item SCS, the scoring ranged from 0.35 to 0.70. These data indicate a high degree of internal consistency for both forms of the SCS (Table 3).

**Table 3:** Internal consistency and test-retest reliability for the long and short forms of the SCS

	**Internal consistency**	**Test-retest**
	**(Cronbach’s α)**	**(ICC)**
Long SCS		
Self-kindness	.79	.88
Self-judgment	.76	.84
Common humanity	.72	.86
Isolation	.77	.81
Mindfulness	.73	.93
Over-identification	.76	.85
Total SCS score	.87	.92
Short SCS		
Self-kindness	.73	.91
Self-judgment	.71	.82
Common humanity	.75	.84
Isolation	.77	.79
Mindfulness	.74	.85
Over-identification	.76	.87
Total SCS score	.85	.89

With regard to temporal stability, a subsample of 112 (41.79%) individuals from sample 1 was randomly selected and a new interview in order to complete the instruments arranged for 1–2 weeks later. In this subsample, 63.39% were female, the mean age was 21.15 (SD = 1.93), 100% were European, 1.7% practiced any type of meditation at least once a month, and most of these individuals (56.25%) studied medicine (57.08%). There were no significant differences in sociodemographic variables between this subsample and the entire sample. In this subsample, the 26-item form of the SCS showed a test-retest coefficient measured with an ICC of 0.92 (95% CI = 0.89–0.94). The ICC for the 6 subscales ranged from 0.84 to 0.93.

Another subsample of 103 (38%) individuals from sample 2 was randomly selected and a new interview arranged for 1–2 weeks later. In this subsample, 69.9% were female, the mean age was 36.81 years (SD = 8.93), 100% were European and only 0.97% practiced any type of meditation at least once a month. Most of these individuals were doctors (66.01%). There were no significant differences in the sociodemographic variables between this subsample and the entire sample. In this subsample, the 12-item form of the SCS showed a test-retest coefficient of 0.89 (CI: 0.87-0.93). The ICC for the 6 subscales ranged from 0.79 to 0.91).

### Construct validity and correlation between both forms and with other associated psychological constructs

To test construct validity, Pearson’s correlation coefficients were calculated between both scales of the SCS and other questionnaires measuring related constructs. The studied constructs follow a normal distribution. As expected, the 26 and 12-item forms of the SCS showed a significant negative correlation with the BDI, STAI and PSQ, and a significant positive correlation with the MAAS (Table 4).

**Table 4:** Correlation between the long and short forms of the SCS and other associated psychological constructs

**Variable**	**Correlation (Pearson’s r)**	
	**26 item SCS**	**12 item SCS**
BDI (Depression)	-.43*	-.38*
STAI-Trait (Anxiety trait)	-.54*	-.49*
PSQ (Perceived stress)	-.58*	-.52*
MAAS (Mindfulness)	.41*	.39*

*p < .01.

Regarding the correlation between the 26 and 12-item forms of the SCS, the following correlations between the corresponding subscales were observed: r =0.89 for Self-Kindness, r =0.90 for Self-Judgement, r =0.81 for Common Humanity, r =0.81 for Isolation, r =0.83 for Mindfulness and r =0.90 for Over-Identification. The correlation between the total score of the long and short forms was high (r =0.92).

Finally, correlations between the subscales of the long and short forms of the SCS and other associated psychological constructs are summarized in Table 5.

**Table 5:** Correlation between the subscales of the long and short forms of the SCS and other associated psychological constructs

	**BDI**	**STAI-trait**	**PSQ**	**MAAS**
*Long SCS*				
Self-kindness	-.40*	-.57*	-.52*	.36*
Self-judgment	.41*	.55*	.54*	-.34*
Common humanity	-.47*	-.49*	-.56*	.38*
Isolation	.45*	.47*	.55*	-.33*
Mindfulness	-.39*	-.52*	-.61*	.50*
Over-identification	.40*	.53*	.60*	-.48*
Total SCS score	-.43*	-.54*	-.58*	.41*
*Short SCS*				
Self-kindness	-.42*	-.52*	-.48*	.36*
Self-judgment	.39*	.54*	.46*	-.33*
Common humanity	-.43*	-.44*	-.49*	.37*
Isolation	.44*	.46*	.47*	-.35*
Mindfulness	-.35*	-.48*	-.55*	.46*
Over-identification	.36	.46*	.53*	-.44*
Total SCS score	-.38*	-.49*	. -.52*	.39*

*p < .01.

## Discussion

The main purpose of the present study was to validate the Spanish versions of the long and short forms of the SCS in university students (long form) and health workers (short form). To our knowledge, despite the importance of the self-compassion construct, no validation of any of the two forms in any other language had been previously performed, apart from the original English version of both scales [11,14] and a Dutch version of the 26 [38] and 12-item forms [14].

In our samples, both forms of the SCS showed high internal consistency, high test-retest reliability and expected and significant correlations with associated psychological variables, such as depression, trait anxiety, perceived stress (negative correlation) and mindfulness (positive correlation). In addition, the results observed using CFA were largely consistent with those reported in previous studies [11,14,38]: the six-factor model of both scales showed adequate fit and all items loaded strongly onto the expected latent factor. This factor structure is maintained cross-culturally [16], thus these results could be expected. Moreover, according to the criteria of Stöber & Joormann [39] for creating short forms of longer self-report measures, the 12-item form of the SCS fulfils both criteria: (a) high correlations with the long SCS and (b) high correlations of the short version subscales with their intended subscales of long form of the SCS.

When we analysed the correlations between the subscales of the long and the short forms of the SCS and the other questionnaires used in the study, we were able to confirm that all of them met our expectations. All correlations were high and some of them, as expected, even more so. For instance, the mindfulness subscale of SCS correlates highly with MAAS, while the isolation subscale of SCS also correlates more highly with BDI. The correlations in this study were similar to those found in the original validation study [10], but the correlation between compassion and mindfulness, despite being high, was lower than that found in other studies [13].”

Unlike mindfulness [3,4], self-compassion is a robust psychological construct reliably measured using SCS. This questionnaire can be used to monitor the effectiveness of MBIs in different fields of clinical psychology, such as personality trait disorders [40,41] or adaptive processes [42].

The short 12-item form of the SCS, as the authors suggest [14], might be particularly useful in settings where time constraints make the use of the long form less feasible or advisable (e.g., time-consuming survey research, therapy process-outcome research and individual treatment monitoring in daily clinical practice). However, these authors recommend using the full scale if information about subscales is crucial [14].

The main limitation of this study is associated with the characteristics of the psychological construct: the validity of self-report measures of mindfulness, the MAAS in particular, has been criticised because the respondents might not be fully aware of their ability to experience the present moment [43]. MAAS only assesses “mindlessness” and not acceptance, a relevant facet in mindfulness and a concept closer to compassion than mindfulness itself. Future studies should study the correlation of compassion with the subscales of FFMQ. Regarding SCS, the original authors [11] acknowledge that a self-report scale will be limited in its ability to accurately assess individual levels of self-compassion, as many people (for example, those who repress or avoid their negative emotions) are aware enough of their own emotional experiences to realise the extent to which they lack self-compassion. Another limitation, as in any study using self-report measures, is that the results might be influenced by participants’ acquiescence and need for social desirability. However, this specific aspect was assessed and ruled out in the original validation study [11]. Moreover, this sample is not representative of the general population, but rather represents a highly educated population. In addition, this sample primarily comprises non-meditators. Thus, it is possible that the self-compassion scale, in a general population sample or a sample of mindfulness meditators, might show different psychometric properties and factor structures, suggesting a relevant research target for future studies. Finally, the fact that both samples comprised healthcare workers, i.e. professionals dedicated to helping others, led us to believe that compassion might be more present in this group in comparison with the rest of the population, and therefore presenting certain bias. Nevertheless, the study of this population was very interesting owing to fact that it presented a high risk of developing burnout syndrome, one of the facets of which is depersonalisation, a construct that is practically antagonistic to compassion.

## Conclusions

The Spanish versions of the 26 (long) and 12 (short)-item forms of the SCS have been validated as reliable instruments for measuring self-compassion in the general population. Although this psychological construct is considered one of the key mindfulness-related positive mental states, there have not been many studies that enhance our knowledge of this concept and assess the outcomes in psychiatric disorders. This study will facilitate an assessment of self-compassion for clinical and research purposes in Spanish-speaking populations.

## Abbreviations

MBIs: Mindfulness-based interventions; SCS: Self-compassion scale; SCS-CH: Common humanity; SCS-M: Mindfulness; SCS-SK: Self-kindness; MAAS: Mindful attention awareness scale; STAI: State-trait anxiety inventory; BDI: Beck depression inventory; PSQ: Perceived stress questionnaire; SD: Standard deviation; CFA: Confirmatory factor analysis; ICC: Intraclass correlation coefficient; CFI: Comparative fit index; GFI: Goodness of fit index; SRMR: Root mean square error of approximation; RMSEA: Standardised root mean-square residual.

## Competing interests

The authors declare that they have no competing interests.

## Authors’ contributions

JGC and MMPD designed the project. MNG, LLA and MMPD collected the data. EA and JMM performed the statistical analysis, and all authors interpreted the results, drafted the manuscript and read and approved the final manuscript.

## Supplementary Material

Additional file 1 **Annex 1.** Spanish version of the 26 item form of the SCS. **Annex 2.** Spanish version of the12 item form of the SCS-SF (Self-Compassion Scale-short form). Click here for file
