# Supplementary material for: Validation of the Spanish versions of the long (26 items) and short (12 items) forms of the Self-Compassion Scale (SCS)
Source: Health Qual Life Outcomes. 2014 Jan 10;12:4. doi: 10.1186/1477-7525-12-4 (PMC3896764; doi:10.1186/1477-7525-12-4)
Supplement: Additional file 1 — Annex 1. Spanish version of the 26 item form of the SCS. Annex 2. Spanish version of the12 item form of the SCS-SF (Self-Compassion Scale-short form). [file 1477-7525-12-4-S1.docx]

Additional file

Annex 1.- Spanish version of the 26 item form of the SCS

¿CÓMO ACTÚO HABITUALMENTE HACIA MÍ MISMO EN MOMENTOS DIFÍCILES?

Lea cada frase cuidadosamente antes de contestar. A la izquierda de cada frase, indique la frecuencia con que se comporta de la manera indicada, utilizando la siguiente escala:

Casi Casi

Nunca Siempre

1 2 3 4 5

_____ 1. Desapruebo mis propios defectos e incapacidades y soy crítico/a respecto a ellos.

_____ 2. Cuando me siento bajo/a de ánimo, tiendo a obsesionarme y a fijarme en todo lo que va mal.

_____ 3. Cuando las cosas me van mal, veo las dificultades como parte de lo que a todo el mundo le toca vivir

_____ 4. Cuando pienso en mis deficiencias, tiendo a sentirme más separado/a y aislado/a del resto del mundo.

_____ 5. Trato de ser cariñoso/a conmigo mismo/a cuando siento malestar emocional.

_____ 6. Cuando fallo en algo importante para mí, me consumen los sentimientos de ineficacia.

_____ 7. Cuando estoy desanimado y triste, me acuerdo de que hay muchas personas en el mundo que se sienten como yo.

_____ 8. Cuando vienen épocas muy difíciles, tiendo a ser duro/a conmigo mismo/a.

_____ 9. Cuando algo me disgusta trato de mantener mis emociones en equilibrio.

_____ 10. Cuando me siento incapaz de alguna manera, trato de recordarme que casi todas las personas comparten sentimientos de incapacidad.

_____ 11. Soy intolerante e impaciente con aquellos aspectos de mi personalidad que no me gustan.

_____ 12. Cuando lo estoy pasando verdaderamente mal, me doy el cuidado y el cariño que necesito.

_____ 13. Cuando estoy bajo/a de ánimo, tiendo a pensar que, probablemente, la mayoría de la gente es más feliz que yo.

_____ 14. Cuando me sucede algo doloroso trato de mantener una visión equilibrada de la situación.

_____ 15. Trato de ver mis defectos como parte de la condición humana.

_____ 16. Cuando veo aspectos de mí mismo/a que no me gustan, me critico continuamente.

_____ 17. Cuando fallo en algo importante para mí, trato de ver las cosas con perspectiva.

_____ 18. Cuando realmente estoy en apuros, tiendo a pensar que otras personas lo tienen más fácil.

_____ 19. Soy amable conmigo mismo/a cuando estoy experimentando sufrimiento.

_____ 20. Cuando algo me molesta me dejo llevar por mis sentimientos.

_____ 21. Puedo ser un poco insensible hacia mí mismo/a cuando estoy experimentando sufrimiento.

_____ 22. Cuando me siento deprimido/a trato de observar mis sentimientos con curiosidad y apertura de mente.

_____ 23. Soy tolerante con mis propios defectos e imperfecciones o debilidades.

_____ 24. Cuando sucede algo doloroso tiendo a hacer una montaña de un grano de arena.

_____ 25. Cuando fallo en algo que es importante para mí, tiendo a sentirme solo en mi fracaso.

_____ 26. Trato de ser comprensivo y paciente con aquellos aspectos de mi personalidad que no me gustan.

Clave de codificación:

Auto-Amabilidad, ítems: 5, 12, 19, 23, 26 Auto-Juicio, ítems: 1, 8, 11, 16, 21

Humanidad común, ítems: 3, 7, 10, 15 Aislamiento, ítems: 4, 13, 18, 25

Mindfulness, ítems: 9, 14, 17, 22 Sobre-identificación, ítems: 2, 6, 20, 24

Cada subescala se calcula realizando la media de las respuestas a todos los ítems de la subescala. Para calcular el valor total de cada una de las 3 subescalas principales (auto-compasión, humanidad común y mindfulness) hay que hacer la media de los ítems de dichas subescalas en forma directa. A esa cifra se le suma la media de los ítems de las subescalas complementarias (de auto-amabillidad es auto-juicio, de humanidad común es aislamiento y de mindfulness es sobreidentificación), que se calculan de forma inversa (ej: 1 = 5, 2 = 4, 3 = 3. 4 = 2, 5 = 1). La media de cada una de las 3 subescalas se calcula, por tanto, haciendo la media de las dos subescalas complementarias, corrigiendo de forma directa la escala principal y de forma inversa la escala complementaria.

Annex 2.- Spanish version of the12 item form of the SCS-SF (Self-Compassion Scale-short form)

CÓMO SUELO ACTUAR CONMIGO MISMO/A EN MOMENTOS DIFÍCILES

Por favor, antes de responder, lea atentamente las siguientes afirmaciones. A la izquierda de cada ítem, indique con qué frecuencia actúa en la forma señalada, empleando la siguiente escala:

Casi Nunca Casi siempre

1 2 3 4 5

_____ SC1. Cuando fallo en algo importante para mí, me consumen los sentimientos de ineficacia.

_____ SC2. Trato de ser comprensivo y paciente con aquellos aspectos de mi personalidad que no me gustan.

_____ SC3. Cuando me sucede algo doloroso trato de mantener una visión equilibrada de la situación.

_____ SC4. Cuando estoy bajo/a de ánimo, tiendo a pensar que la mayoría de la gente es probablemente más feliz que yo.

_____ SC5. Trato de ver mis defectos como parte de la condición humana.

_____ SC6. Cuando lo estoy pasando verdaderamente mal, me doy el cuidado y el cariño que necesito.

_____ SC7. Cuando algo me disgusta, trato de mantener mis emociones en equilibrio.

_____ SC8. Cuando fallo en algo que es importante para mí, tiendo a sentirme solo en mi fracaso.

_____ SC9. Cuando me siento bajo/a de ánimo, tiendo a obsesionarme y fijarme en todo lo que va mal.

_____ SC10. Cuando me siento incapaz de alguna manera, trato de recordarme que casi todas las personas comparten sentimientos de incapacidad.

_____ SC11. Desapruebo mis propios defectos e incapacidades y soy crítico/a respecto a ellos.

_____ SC12. Soy intolerante e impaciente con aquellos aspectos de mi personalidad que no me gustan.

Clave de codificación:

Auto-Amabilidad, ítems: 2, 6 Auto-Juicio, ítems: 11, 12

Humanidad común, ítems: 5, 10 Aislamiento, ítems: 4, 8

Mindfulness, ítems: 3, 7 Sobre-identificación, ítems: 1, 9

Cada subescala se calcula realizando la media de las respuestas a todos los ítems de la subescala. Para calcular el valor total de cada una de las 3 subescalas principales (auto-compasión, humanidad común y mindfulness) hay que hacer la media de los ítems de dichas subescalas en forma directa. A esa cifra se le suma la media de los ítems de las subescalas complementarias (de auto-amabillidad es auto-juicio, de humanidad común es aislamiento y de mindfulness es sobreidentificación), que se calculan de forma inversa (ej: 1 = 5, 2 = 4, 3 = 3. 4 = 2, 5 = 1). La media de cada una de las 3 subescalas se calcula, por tanto, haciendo la media de las dos subescalas complementarias, corrigiendo de forma directa la escala principal y de forma inversa la escala complementaria.
